# Effects of composition on catalytic activities of molybdenum doped platinum nanoparticles

**DOI:** 10.3906/kim-2001-63

**Published:** 2020-08-18

**Authors:** Aslıhan SÜMER

**Affiliations:** 1 Faculty of Pharmacy, University of Health Sciences Turkey, İstanbul Turkey

**Keywords:** Carbon monoxide, nanoparticle, catalysis, platinum, molybdenum

## Abstract

The physical and chemical properties of bimetallic nanoparticles can be optimized by tuning the particle composition. In this study, we identified CO adsorption and dissociation energetics on five Pt-Mo nanoparticles at different concentrations, the lowest energy Pt_7_, Pt_6_Mo, Pt_5_Mo_2_, Pt_4_Mo_3_, and Mo_7_ clusters. We have shown that the CO adsorption and dissociation energies and preferred CO adsorption sites are largely dependent on the composition of the nanoparticles. As the Mo concentration increases, the strength of the C-O internal bond in the adsorption complex decreases, as indicated by a decrease in the C-O stretching frequency. Also, more Mo sites in the nanoparticle become available for CO adsorption, and the preferred CO adsorption site switches from Pt to Mo. For these reasons, dissociation of CO is energetically favorable on Pt_4_Mo_3_ and Mo_7_. On both compositions, we have shown that the dissociation paths begin with CO adsorbed on a Mo site in a multifold configuration, in particular in a tilted configuration. These findings provide insight on the effects of the composition on the chemical and catalytical properties of Pt-Mo nanoparticles, thereby guiding future experiments on the synthesis of nanoparticles, especially those that may be suitable for various desired applications containing CO.

## 1. Introduction

Atomic and molecular nanoparticles and related systems and phenomena are subjects of a rapidly developing research field. The ever-growing interest in nanoparticles reflects the central role these systems play in different technologies (e.g., catalysis, medicine, thin films, coatings, and others) [1–5]. The unique potential of nanoparticles to attain various thermal properties, electronic features, and chemical reactivity through changes in size, structure, and composition allows researchers to customize them according to desired application areas [6–9].

Catalysis is one of the significant areas where nanoscience finds application. Increased surface/volume ratio of nanocatalysts in small sizes offers the opportunity to reduce the consumption of expensive raw materials. Also, by tuning size, shape, and composition, it is possible to achieve high activity and selectivity in chemical transformations. One of these reactive systems where nanocatalysts find application is at the anodic side of low-temperature fuel cells [10]. Pure Pt has a high activity as an anode catalyst, but it suffers from what’s called CO poisoning. Due to the formation of strong Pt-CO bonds, the active sites are blocked even in the presence of small amounts of CO in the fuel gas and thus, the hydrogen dissociation performance of the catalyst degrades rapidly. A large number of studies have shown that Pt-Mo bimetallic nanoparticles have better anodic activity in the presence of CO than Pt nanoparticles. [11–13]. Auger electron spectroscopy, low energy electron diffraction, and temperature-programmed desorption measurements showed that CO adsorption is weaker on a single Pt monolayer deposited on Mo(110) compared to both pure Pt(111) and Mo(110). Another finding in this study is that deposition of a monolayer of Pt eliminates the dissociative adsorption of CO on Mo(110). As the coverage of Pt on Mo(110) increases, the adsorption energy of CO approaches that of Pt(111). However, more than one Pt monolayer is thermally unstable and agglomerates into three-dimensional (3D) clusters upon heating [14]. A computational study with density functionals proved that the CO adsorption strength is indeed lower on Pt_2_Mo(111) than on pure Pt(111) and that CO is more stable atop the Mo site than it is atop the Pt site on the mixed surface [15].

Because of their relative simplicity, research studies on larger size metal nanocatalysts and their extended surfaces are, in general, abundant. The overall complexity of smaller size nanoparticles with various structural forms and reactive sites makes it challenging to explore these systems. In a study on small size pure Pt nanoparticles, using temperature-programmed reaction and Fourier transform infrared spectroscopy, Heiz et al. showed that the oxidation rate of CO on Pt20 was higher than on Pt_8_ [16]. Gruene et al. studied C-O stretching frequency on pure Pt clusters with 3 to 25 atoms by infrared multiple photon dissociation spectroscopy and found that the preferred CO adsorption configuration was atop, regardless of the size [17]. In a recent study with mass spectrometry, IR photodissociation spectroscopy, and density functional theory (DFT), it was found that a single Mo dopant atom in gas-phase Pt^+^_n_ (n = 3–14) clusters reduces CO binding energies [18]. There is still room for work on the reactivity of small size pure Pt and Mo and bimetallic Pt-Mo nanoparticles towards CO. Particularly the relations between composition and reactivity need to be more thoroughly investigated to design novel nanocatalysts for CO-containing reaction systems.

In this study, we explored the reactivity of 7-atom pure and mixed Pt_7-n_ Mon (n = 0–3,7) clusters towards CO (within the low CO coverage limit) via computations within the framework of density functionals. The number of structural isomers, possible CO adsorption sites and CO dissociation pathways increase with the size of the cluster. To keep the computational cost reasonable, it is essential to use the minimum cluster size that can represent the composition induced changes in the reactivity of clusters towards CO. The energetically most preferred conformation of Ptn changes from planar to 3D structure at n = 7 [19]. Thus, the smallest 3D Pt cluster, Pt_7_, was chosen as a model to represent the typical platinum cluster in this study. We started the work with an initial thorough search of the most accurate computational parameters using existing experimental data. We then determined the energetically most stable isomeric forms of Pt_7-n_Mo_n_, placed a CO molecule on every possible adsorption site of Pt_7-n_Mo_n_, optimized these CO-Pt_7-n_Mo_n_ adsorption complexes, and calculated CO adsorption/desorption energies. In the next step, starting from the stable CO-Pt_7-n_Mo_n_ adsorption complexes, we identified the minimum energy pathways for the CO dissociation reactions, i.e. for the breaking of C-O bond. We calculated the energy barriers on each path so that we identified the energetically most favorable dissociation path for each composition. Finally, we correlated these findings about CO reactivity with the electronic structure of Pt-Mo nanoparticles.

## 2. Methodology

### 2.1. Computational parameters

DFT computations were performed with NWChem 6.0 quantum chemistry package [20] and PW91 [21] exchangecorrelation functional. The exchange-correlation potential was integrated on a fine grid. Effective core potential based spherical Gaussian basis sets with the (8s,7p,6d,1f)/[4s,4p,3d,1f] contraction scheme, denoted as cc-pVDZPP [22,23], were used for Pt and Mo. The effective core potentials explicitly treat 18 and 14 valance electrons for Pt and Mo, respectively. For C and O atoms, 6-311G(2df,2pd) basis set was used. To speed up convergence, smearing was used. At the initial stages of the geometry optimization, a smearing value of 0.005 au was applied. This was decreased to 0.0003 au at the final stages of the optimization to ensure integer occupation of molecular orbitals. The computations were all performed in a spin unrestricted fashion, and the multiplicity was not fixed. This allowed the system to converge to the optimum spin state with the help of smearing. In order to verify that the obtained spin is indeed the best, the achieved geometry was then re-optimized by fixing the spin to a higher and lower value of the one obtained with smearing. A vibrational frequency analysis based on harmonic approximation was also performed on the CO-metal adsorption complex to ensure that the true electronic minimum was reached.

The initial geometric coordinates used to sample the potential energy surface of pure metal cluster configurations were mainly obtained from literature. Initial mixed structures were mainly generated from these pure structures by replacing the symmetry-unique Pt (Mo) atoms in the Pt_7_(Mo_7_) with Mo (Pt) atoms in a systematic fashion. The details are given in the supplemental material. At the end of a geometry optimization, to evaluate the stability of a Pt_7-n_Mo_n_ cluster, binding energy (BE) was calculated as follows:

(1)BE=[7-n) x EPt+ n x EMo-Ecluster]/7,

where E^Pt^, E^Mo^, and E^cluster^ are energies of, respectively, Pt atom, Mo atom, and Pt_7-n_Mo_n_ cluster. BE is a positive number and a higher BE means a more stable cluster with respect to the individual constituent metal atoms.

The adsorption energy of CO (E_Ads_) was computed as follows:

(2)EAds=(Ecluster+ECO-Etotal),1

where E^CO^ and E^total^ are the energies of gas phase CO and CO-Pt_7-n_Mo_n_ adsorption complex, respectively.

### 2.2. Identification of transition states

**Figure 1:**
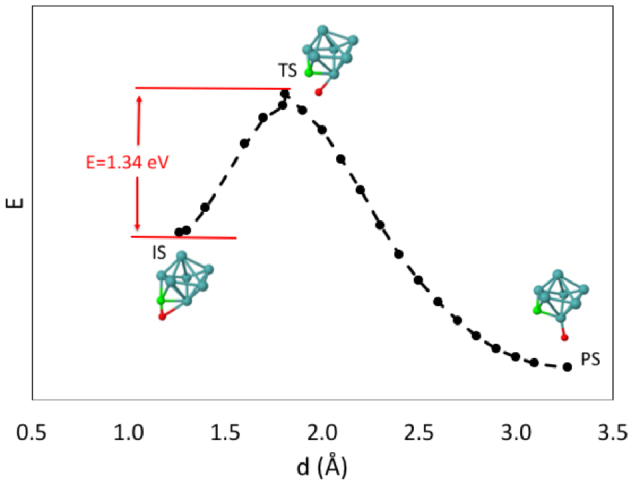
Calculated minimum energies for CO-Mo_7_ as a function of C-O bond distance, d, mapped out by constrained structural energy minimizations along grids of fixed C-O distances. Final transition energy (TS) is obtained by applying eigenvector following procedure to the highest energy CO-Mo_7_ configuration. Initial CO adsorption (IS) and final product (PS) states are also given. The calculated activation energy for C-O breaking is shown in red.

In order to identify the energetics of CO dissociation, we mapped out the complete minimum energy paths to separate an O atom from C on the potential energy surface by performing constrained structural energy minimizations along the grids of fixed C-O distances. The step size along the grids was 0.1 Å. Energy values as a function of the C-O bond distance obtained as such on Mo7 are given as an example in Figure 1. The final refinement of transition states was carried out by applying eigenvector following method as implemented in NWChem to the highest energy configuration of each grid-based minimum energy path [24]. The transition states determined in this way were then verified by normal mode analysis. The connectivity of the transition states to the reactants and to the products was checked by taking small initial steps away from the stationary point and then performing a complete geometric relaxation. All possible stable CO adsorption conformations were considered as initial states for the characterization of C-O bond breaking pathway.

### 2.3. Verification of computational framework

The computational parameters, including the exchange-correlation functional, the basis set/pseudopotential, and energy convergence criteria, have been chosen based on extensive tests performed on Pt and Mo atoms, Pt_2_ and Mo_2_ dimers, and gas phase CO molecule, using alternative choices and comparing the calculated results with the available experimental data (Table 1).

**Table 1:** Computed versus measured properties of single Pt and Mo atoms and Pt_2_ and Mo_2_ dimers: ionization potential (IP), electron affinity (EA), average binding energy (BE), metal-metal bond distance (d) and metal-metal stretching frequency (ν) .

	Mo		Pt	
	Computed	Experimental	Computed	Experimental
d_*dimer*_ (Å)	1.99	1.94 [25]	2.35	2.33 [31]
BE_*dimer*_ (eV)	3.78	4.12+/-0.65 [26]	3.62	3.14-3.71 [32,33]
ν_*dimer*_ (cm^-1^)	380	476 [27]	231	222 [34]
IP_*atom*_ (eV)	7.58	7.22 [28]	8.69	8.96 [35]
IP_*dimer*_ (eV)	7.11	6.95 [29]	8.70	8.68 [32]
*EA_*atom*_ (eV)	0.52	0.75 [30]	2.17	2.12-2.13 [36,37]
*EA_*dimer*_ (eV)	0.80	-	2.19	1.90 [38]

*Calculated with diffuse functions in the basis set (aug-cc-pVDZ).

The ground spin state of the neutral Mo atom (s^1^d^5^) is a septet. Mo_2_ dimer is antiferromagnetic and is at singlet spin state. It has a bond length of 1.99 Å and BE of 3.78 eV. The experimentally measured bond length and BE are 1.94 Å [25] and 4.12+/-0.65 eV [26], respectively. Although the computed and experimentally measured values of BE and bond length are close to each other, there is a significant difference in frequency: The computed frequency of the Mo_2_ dimer is 380 cm^-1^, it is smaller than the experimental value of 476 cm^-1^ [27].

Mo cation and anion have both sextet spin states. The ionization potential (IP) of the Mo atom is found as 7.58 eV and is close to the experimentally measured value, 7.22 eV [28]. The adiabatic IP of the dimer is calculated as 7.11 eV, which is also very close to the experimentally determined IP value of 6.95 eV [29]. The electron affinities (EA) of the Mo atom and dimer are 0.52 eV and 0.80 eV, respectively. The experimentally measured EA of the Mo atom is 0.75 eV [30]. There is no experimental EA value for the dimer.

The optimum spin states of the neutral Pt atom (s^1^d^9^) and Pt_2_ dimer are both triplets. The Pt-Pt bond length of the dimer is 2.35 Å, very close to the experimentally measured value of 2.33 Å [31]. BE is 3.62 eV/atom.Experimental values for BE range from 3.14 +/- 0.02 eV, according to the two-photon ionization experiments[32], to 3.71 eV +/- 0.16 eV, according to the high-temperature equilibrium studies [33]. The variation inexperimentally measured BE makes it difficult to draw a definite conclusion. The computed frequency of Pt_2_ dimer is 231 cm^-1^, very close to the experimental value, 222 cm^-1^ [34].

Pt cation and anion have both doublet spin states. IP and EA are computed as 8.69 and 2.17 eV, respectively. Experimental IP and EA values are 8.96 eV [35] and 2.123–2.125 eV [36,37], respectively. Pt_2_ cation and anion have quartet spin states. The IP and EA of Pt2 are 8.70 and 2.19 eV, respectively. The experimental values for these two quantities are 8.68 ±0.02 eV [32] and 1.898 ±0.008 eV [38]. The calculatedIP and EA correlate very well with the experimental values for the Pt atom and dimer.

We also tested some of the most commonly used hybrid functionals in the literature, i.e. X3LYP, B3LYP, PBE0, on the properties of single Pt and Mo atoms and Pt_2_ and Mo_2_ dimers. The three hybrid functionals,X3LYP, B3LYP, and PBE0 give a BE of 2.60, 2.63 and 2.26 eV for Pt_2_, and 2.88, 2.90, and 2.07, for Mo_2_, respectively. Low BE values calculated compared to experimental values prove that hybrid functionals are notsuitable for use in transition metal computations. This was previously shown by Apra and Fortunelli withcuboctahedral Pt13 and Pt55 clusters [39] and by Du et al. for eight other transition metal dimers [40].

The gas-phase CO molecule is a singlet with a bond length of 1.13 Å. Its BE is calculated as 11.76 eV, which is close to the experimental value, 11.1–11.2 eV [41,42]. The C-O vibrational frequency is computed as 2142 cm^-1^. Its IP is 13.88 eV. Both of these values are very close to the experimental values, 2170 cm^-1^ [41] and 14.01 eV [43], for frequency and IP, respectively.

For CO adsorbed on a single Mo atom (S = 2), E_Ads_ and C-O stretch vibrational frequency are found as 1.05 eV and 1890 cm^-1^, respectively. Hossain and Jarrold computed these two values with B3LYP as 0.94 eV and 1945 cm^-1^ [44]. E_Ads_ on the Mo_2_ dimer is 0.97 and 0.94 eV, for atop (S = 0) and bridge (S = 0) type adsorption configurations, respectively. The C-O stretching frequencies are 1951 and 1734 cm^-1^, for atop and bridge adsorption, respectively. We also computed the energy of CO desorption from Mo(CO)_6_ and MoCO^+^ complexes. Lewis et al. [45] measured the gas phase first CO desorption energy from Mo(CO)6 as 1.75 eV with laser pyrolysis. Michels et al. [46] measured the desorption energy from MoCO^+^ as 1.72 eV. We calculated CO desorption energies from Oh -symmetric Mo(CO)_6_ and from MoCO^+^ as 1.87 and 1.71 eV, respectively, which are very well correlated with the experimental values.

The CO adsorbed on a single Pt atom (S = 0) has a vibrational C-O stretching frequency of 2066 cm^-1^. This computed value is very close to the experimentally measured value of 2070 cm−1 [17]. Manceron et al. computed E_Ads_ as 3.36-3.39 eV using MP2 level of theory and different basis sets [47]. We computed E_Ads_ as 3.73 eV, which is slightly higher than the calculated values with MP2. On the Pt_2_ dimer, E_Ads_ values for atop and bridge type adsorption configurations are 2.41 and 3.06 eV, respectively. The C-O stretching frequencies are 2037 and 1839 cm^-1^, for atop (S = 1) and bridge (S = 0) adsorption configurations, respectively. Roszak and Balasubramanian found frequencies as 2029 and 1887 cm^-1^, for atop and bridge adsorption, respectively, with MP2 [48].

Basis set superposition errors (BSSE) in the adsorption energy of CO on single metal atoms and dimers were computed with the counterpoise method implemented in NWChem. E_Ads_ of CO on the Pt atom decreases by 0.057 eV after BSSE correction. E_Ads_ of CO on the Pt_2_ dimer decreases by 0.139 and 0.094 eV, for atop and bridge type adsorption configurations, respectively. On the Mo atom, BSSE correction decreases E_Ads_ by 0.055 eV. On the Mo_2_ dimer, E_Ads_ decreases by 0.062 and 0.085 eV, for atop and bridge type adsorption configurations, respectively. Considering that the effect of BSSE is relatively low and does not vary greatly with size and adsorption configuration, we neglected the effect of BSSE on larger size structures.

## 3. Results

### 3.1. Energetically most stable Pt-Mo nanoparticles at different compositions

The most stable 7-atom pure Pt and Mo clusters (with the highest BE) along with bimetallic Pt-Mo clusters are shown in Table 2, together with their spin states, symmetries, and average binding energies. The most stable Pt7 conformation is in agreement with that of Chaves et al. [49]. Its optimum spin state is quintet. The 3-dimensional packing of this lowest energy isomer contradicts previous studies claiming that Pt clusters prefer planar packing up to n = 9 [50]. A recent scanning tunneling microscopy study on small size Pt/TiO2 clusters shows that the transformation from planar to 3-dimensional packing in Ptn nanoparticles occurs at size n = 8 atoms [51]. However, the metal clusters in this study are under the influence of strong metal-support interactions, and the findings might not precisely reflect the gas-phase structural properties of Ptn . The lowest energy structure for Mo7 is at triplet spin state. It was also predicted by Zhang et al. [52] and Ziane et al. [53]. A structure with the same overall packing but at singlet spin state was identified as the lowest energy structure by Min et al. [54]. We found that the singlet state of Mo7 is 0.015 eV higher in energy than the triplet state. Using a different exchange-correlation functional (PBE at the place of PW91) and projector augmented wave pseudopotentials may cause the singlet ground state in reference 54. The selection of the exchange-correlation functional and basis set in DFT calculations can have a strong influence on the energy order between different spin states, particularly when the energies of the states are close. It is therefore crucial to test the agreement between calculated results and the available experimental data to verify the choice of computational details, and in particular the choice of the exchange-correlation functional, whenever possible.

**Table 2:** Low energy Pt_7-n_ Mon (n = 0–3 and 7), their symmetry group (Sym.), spin state (S) and average binding energy in eV/atom (BE). (Pt, Mo, C and O)

Composition	Pt_7_	Pt_6_Mo	Pt_5_Mo_2_	Pt_4_Mo_3_	Mo_7_
	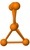	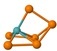	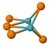	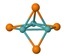	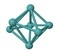
Sym	C_2_	C_s_	C_2v_	C_s_	C_s_
S	2	1	1	0	1
BE (eV/atom)	3.26	3.64	3.77	3.81	3.35

In the most energetically stable bimetallic Pt-Mo clusters, Mo atoms are located in the center of the cluster, while Pt atoms surround Mo atoms in a capping fashion. When the number of Mo atoms is more than one, these Mo atoms prefer clustering instead of dispersing in the nanoparticle. The lowest energy Pt_6_Mo and Pt_5_Mo_2_ are at triplet states and Pt4 Mo3 is at singlet spin state. The average binding energies of mixed clusters are higher than that of both pure Pt and Mo clusters, indicating that mixing has a stabilizing effect on both Pt and Mo and is energetically favored.

### 3.2. CO adsorption on Pt-Mo nanoparticles at different compositions

To determine the stable CO adsorption complexes, we placed a CO molecule on every possible adsorption site of the lowest energy Pt_7-n_Mo_n_ (n = 0–3,7) and optimized these CO-Pt_7-n_Mo_n_ adsorption complexes. Then we calculated CO adsorption/desorption energies. In Table 3, the most stable CO adsorption configurations (i.e. those with highest CO adsorption energies) are given, together with adsorption energies and spin states. Other stable adsorption structures are given in the supplemental material. The preferred adsorption configuration on Pt7 is atop. The adsorption complex is at triplet spin state. Infrared spectroscopy measurements also showed that CO was adsorbed on Pt7 with atop-Pt configuration and a C-O stretching frequency of ~2025 cm^-1^ [17]. Our computed value for C-O stretching frequency, 2042 cm^-1^, is very close to the experimentally determined value.

**Table 3:** CO-Pt_7-n_Mo_n_ (n = 0–3 and 7) with the highest CO adsorption energies, their spin states (S), CO adsorption energies (E_Ads_), C-O bond distances (d_C-O_) and C-O stretching frequencies (ν_C-O_) (Pt, Mo, C and O)

Composition	Pt_7_	Pt_6_Mo	Pt_5_Mo_2_	Pt_4_Mo_3_	Mo_7_
	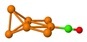	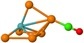	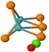	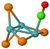	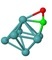
S	1	0	1	0	1
E_Ads_ (eV)	2.58	2.68	1.92	2.03	2.60
d_C-O_ (Å)	1.16	1.16	1.16	1.16	1.26
ν_C-O_ (cm^-1^)	2042	2038	2027	1963	1388

On Mo_7_, CO is adsorbed on a threefold site. In this tilted configuration, C is bonded to a threefold site, while O atom is also bonded to one of the atoms of the same threefold site. C-O bond in the adsorption complex is 1.26 Å, longer than both the one in CO-Pt7 complex (1.16 Å) and also the bond of CO in the gas phase. The C-O stretching frequency in CO-Mo_7_ is significantly smaller than the one in CO-Pt_7_. The differences in the bond lengths and frequencies between Mo-coordinated and Pt-coordinated CO can be explained based on the Blyholder adsorption model. Blyholder defines the CO binding to the metal as a process accompanied by electron “donation” from CO-5σ (HOMO) orbital to metal empty states and “backdonation” from metal occupied states to CO-2π (LUMO) orbital. Electron donation is favored when the adsorption is in atop configuration while backdonation is favored in the multifold adsorption configuration. As CO-2π is C-O antibonding, electron backdonation results in an increase in C-O bond length and a decrease in C-O stretching frequency. From this point of view, electron backdonation is more favored in the multifold adsorption configuration on Mo7 than atop adsorption on Pt_7_. The tilted-adsorbed CO was also identified both experimentally and computationally on extended Mo surfaces. These structures are considered as precursors of CO dissociation at low CO surface coverages [55–59].

On Pt_6_Mo, Pt_5_Mo_2_ and Pt_4_Mo_3_ clusters, the most stable adsorption sites are all atop. However, on both Pt_6_Mo and Pt_5_Mo_2_, CO is adsorbed atop-Pt, while on Pt_4_Mo_3_, CO is adsorbed atop-Mo. As was previously mentioned, Mo atoms prefer to be located at the core of clusters while Pt atoms occupy less coordinated sites. It can be assumed that this atom orientation in the metal cluster and steric effects during CO adsorption play a role in the relative instability of Mo-bonded CO configurations for Pt_6_Mo and Pt_5_Mo_2_ clusters. Lower coordinated Mo atoms that can bind ligands without steric repulsion only become available when the number of Mo atoms in the clusters is high enough.

Composition and isomeric form are among the most important properties of the bimetallic nanoparticles that affect the CO adsorption strength. The composition effect can only be evaluated comparatively if the isomeric form is the same at all compositions. Lordeiro et al. introduced the term “composomers” to refer to these compositional isomers, i.e., clusters with the same number of atoms and geometrical (skeletal) structure but different compositions [60]. In our study, the lowest energy Pt_7-n_Mo_n_ isomers (n = 0–3 and 7) used for CO adsorption all have different isomeric forms. The differences in reactivity of these clusters towards CO are due to the differences in their composition as well as the different geometrical arrangements of atoms in them. This may be one of the reasons why there is no simple relationship between CO adsorption energies and n in the most stable CO-Pt_7-n_Mo_n_ (Table 3). CO adsorption energies on the most stable sites of Pt_5_Mo_2_ (Eads = 1.92 eV) and Pt_4_Mo_3_ (E_ads_ = 2.03 eV) clusters are significantly smaller than on the most stable sites of pure Pt_7_ (E_ads_ = 2.58 eV) and Mo_7_ (E_ads_ = 2.60 eV) clusters. CO adsorption energy on the most stable adsorption site of Pt_6_Mo (Eads = 2.68 eV) cluster, on the other hand, is slightly higher than that on pure clusters. However, if we compare the adsorption energies of all possible adsorption sites (supplemental material) at each composition, it can be concluded that the average adsorption energy is smaller on the mixed clusters, in general, than on the pure ones.

### 3.3. CO dissociation on Pt-Mo nanoparticles at different compositions

**Figure 2:**
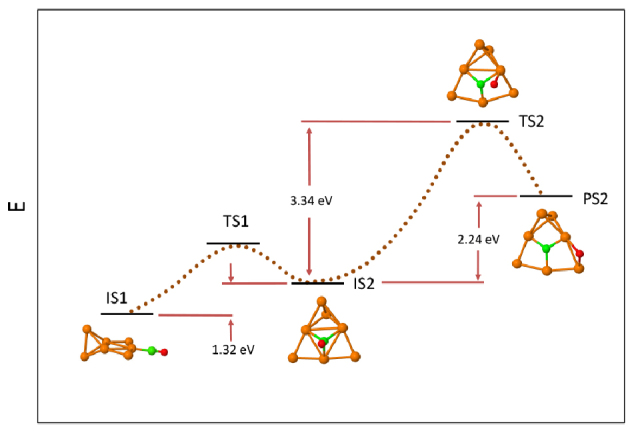
CO dissociation paths for CO-Pt_7_. The path with the lowest energy barrier to dissociation is given, starting from the lowest energy adsorption complex. Energies of initial (IS), transition (TS) and product states (PS) are given. The dotted lines that connect IS, TS and PS are for visual purposes and do not represent real points in the potential energy surface.

**Figure 3:**
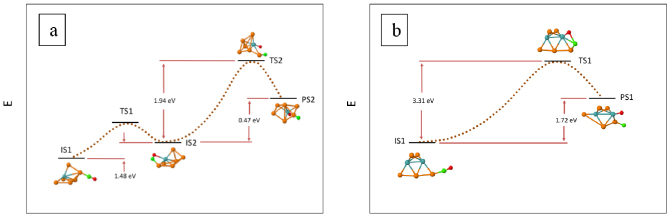
CO dissociation paths for (a) CO-Pt_6_Mo and (b) CO-Pt_5_Mo_2_. The paths with the lowest energy barrier to dissociation at each composition is given, starting from the lowest energy adsorption complex. Energies of initial (IS), transition (TS) and product states (PS) are given. The dotted lines that connect IS, TS and PS are for visual purposes and do not represent real points in the potential energy surface.

**Figure 4:**
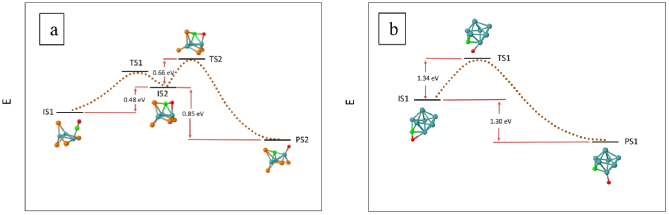
CO dissociation paths for (a) CO-Pt_4_Mo_3_ and (b) CO-Mo_7_. The paths with the lowest energy barrier to dissociation at each composition is given, starting from the lowest energy adsorption complex. Energies of initial (IS), transition (TS) and product states (PS) are given. The dotted lines that connect IS, TS and PS are for visual purposes and do not represent real points in the potential energy surface.

Minimum energy paths for CO dissociation on CO-Pt_7-n_Mo_n_ (n = 0–3 and 7) were found starting from stable adsorption complexes as initial state configurations (IS). Transition (TS) and product (PS) state configurations were identified in the pathways. The energies of the most favorable dissociation pathways with the lowest energy barriers in each composition are given in Figures 2–4 (The other paths with higher barriers can be found in the supplementary material.) In the cases of CO-Pt_7_, CO-Pt_6_Mo and CO-Pt_4_Mo_3_, the IS of the dissociation reaction differs from the energetically most stable adsorption configuration. In Figures 2–4, the energies of the most stable adsorption configurations for these compositions are also shown.

On Pt7 , the initial state (IS2) of the reaction pathway with the lowest energy barrier is a threefold adsorbed CO configuration, as shown in Figure 2. Starting from the lowest energy adsorption complex, IS1 (Table 3), CO first diffuses from IS1 to IS2. The energy of the diffusion is 1.32 eV. The transition state, TS2, for C-O bond breaking, is 3.34 eV higher in energy than IS2. Assuming that the barrier for diffusion is relatively low, then, starting from IS1, the total energy required to overcome in dissociation equals to 4.66 eV. The overall energy of the dissociation, i.e. the energy difference between the final PS2 and IS1 equals to 3.54 eV. The high barrier, as well as the endothermicity of the reaction, indicates that dissociation is not favorable on the pure Pt nanoparticle. Considering that the adsorption energy on IS1 is 2.58 eV, it is concluded that CO molecule prefers to desorb rather than dissociate. This finding is in correspondence with the fact that there is no experimentally verified CO dissociation activity on Pt nanoparticles.

CO dissociation pathways for CO-Pt_6_Mo and CO-Pt_5_Mo_2_ are given in Figure 3. On Pt_6_Mo, CO first diffuses from the lowest energy atop-Pt adsorption configuration (IS1) to a tilted configuration (IS2). The energy of diffusion is 1.48 eV. During C-O bond breaking, the transition state, TS2, has a Pt-bonded C atom, and Mo-bonded O atom. The energy difference between TS2 and IS2 is 1.94 eV. Thus, the total energy required starting from IS1 to TS2 is 3.42 eV. Because the adsorption energy of CO in IS1 is 2.68 eV and the overall heat of dissociation reaction is 1.95 eV, the reaction is kinetically and thermodynamically favorable over desorption on Pt6 Mo. On the mixed Pt5 Mo2 cluster, the C-O bond breaking pathway with the lowest energy barrier starts with IS1, which is also the lowest energy adsorption complex given in Table 3. The transition state, TS1, has a bridge PtMo-bonded C atom and Mo-bonded O atom. The barrier in dissociation is 3.31 eV, and the reaction energy is 1.72 eV. Considering that the adsorption energy of CO in IS1 is 1.92 eV, it can be concluded that dissociation is not favorable over desorption on Pt_5_Mo_2_, just like on Pt_6_Mo.

CO dissociation pathways for CO-Pt_4_Mo_3_ and CO-Mo_7_ are given in Figure 4. On Pt_4_Mo_3_, CO first diffuses from the lowest energy atop adsorption configuration (IS1) to a tilted configuration (IS2). The energy of diffusion is 0.48 eV. During C-O bond breaking, the transition state, TS2, has a trifold PtMo2-bonded C atom, and Mo-bonded O atom. The energy difference between TS2 and IS2 is only 0.66 eV. The total energy required starting from IS1 to TS2 is 1.15 eV. Because the adsorption energy of CO in IS1 is 2.03 eV and the overall heat of dissociation reaction is –0.37 eV, the reaction is kinetically and thermodynamically favorable over desorption on Pt_4_Mo_3_. On Mo_7_, the C-O bond breaking pathway with the lowest energy barrier starts with IS1. This configuration is the lowest energy adsorption complex with a tilted CO given in Table 3. The transition state, TS1, is 1.34 eV higher in energy than IS1. The dissociation reaction is exothermic, and the energy of reaction, i.e., the energy difference between the product PS1 and IS1 is –1.30 eV. The barrier for dissociation is lower than the adsorption energy of CO in the initial state, which is 2.60 eV. Thus, as with Pt_4_Mo_3_, CO dissociation is kinetically and thermodynamically favorable over desorption on Mo_7_. This finding is in parallel to the previous experimental findings on extended Mo surfaces with tilted adsorbed CO at low coverage.

Calculations showed that CO dissociation energies on 7-atom nanoparticles show a strong dependency on the particle composition. For the five metal clusters tested here, CO dissociation-on-particle is favored over desorption-from-particle on Pt_4_Mo_3_ and on Mo_7_. On both of these clusters, the dissociation proceed on multifold Mo sites. The CO stretching vibrations in Table 3 indicate a weakening effect of Mo on CO internal bond. There is a gradual decrease in the stretching frequency of atop-Pt adsorbed CO on Pt_7-n_Mo_n_ as n increases from 0 to 2. A more pronounced decrease is observed when n increases further from 2 to 3 and the preferred adsorption site is changed from atop-Pt to atop-Mo. Overall these findings indicate that the presence of available Mo sites for CO bonding in the cluster is a necessary condition for CO dissociation.

### 3.4. Electronic structure of transition states for CO-Pt_2_ vs. CO-PtMo

To investigate the causes behind reduced energy barriers in CO dissociation on Mo-doped Pt nanoparticles, we analyzed the electronic structure of CO during dissociation on pure Pt2 and mixed PtMo dimers. The small size of the dimers makes it easier to compare the molecular orbitals in different compositions and understand the effect of composition on CO dissociation. To further simplify the analysis, we identified a reaction path in which, independent of the composition of the cluster, the orientation of the CO relative to metal atoms was similar throughout the C-O bond breaking. On Pt_2_, starting from an initial tilted CO adsorption configuration (spin S = 0), the barrier of C-O breaking is calculated as 3.62 eV. On PtMo, similarly starting from an initial tilted adsorption configuration (albeit with spin S = 2), the energy barrier of C-O breaking is 1.81 eV. As observed previously with seven atom nanoparticles, substitution of a Pt atom with Mo reduces the reaction barrier on the dimers.

**Figure 5:**
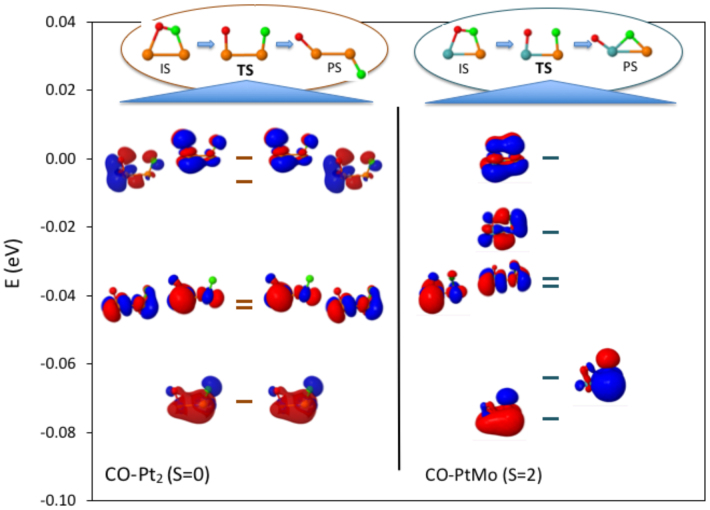
Energy of occupied orbitals of the CO-Pt_2_ (left) and CO-PtMo (right) complexes at the transition states (TS) of CO dissociation. The energy of HOMO (shifted to 0 eV) and energies of other orbitals with respect to HOMO are depicted with horizontal lines. Alpha and beta orbitals are on the left and right sides of the energy lines, respectively. There are 10 (5 alpha + 5 beta) and 6 (5 alpha + 1 beta) molecular orbitals in CO-Pt_2_ and CO-PtMo, respectively. There are four less occupied orbitals in CO-PtMo as compared to CO-Pt_2_, due to the difference in the number of electrons in Pt and Mo atoms.

In Figure 5, IS, TS, and PS of the CO dissociation paths are given, together with molecular orbitals of TS for the two CO-dimer complexes. We plotted the wave functions of the high energy occupied orbitals (with the highest energy occupied orbital shifted to 0 eV), as the differences in the electronic structure between two compositions are more pronounced in this region. A visual analysis of the charge distribution in Figure 5 indicates that in CO-Pt_2_, the two highest energy orbitals in both the alpha and beta manifolds all contribute to C-O bonding. The other two pairs of orbitals with lower energies are mainly metal-metal antibonding, while another pair even lower in energy seems to be metal-metal bonding. On the other hand, of the six orbitals of CO-PtMo given in Figure 5, only two contribute to C-O bonding. These are namely the HOMO and HOMO-1. Two other orbitals lower in energy are mainly metal-metal antibonding, and another pair lowest in energy seems to be metal-metal bonding. Overall, the main difference in Figure 5 which can be related to the higher energy barrier in CO-Pt_2_ (as compared to CO-PtMo) is the relatively higher number of C-O bonding orbitals in this complex. As these bonding orbitals are affected by the increase in the distance between the C and O atoms, it can be assumed that their number can provide an estimate of the energy cost of electronic charge redistribution while the system proceeds from IS to TS.

## 4. Conclusions

We have determined CO adsorption and dissociation energies on five nanoparticles in different compositions: the lowest energy Pt_7_, Pt_6_Mo, Pt_5_Mo_2_, Pt_4_Mo_3_, and Mo_7_ clusters. Both the adsorption and dissociation energies show a dependence on the nanoparticle composition. Comparison of adsorption energies and dissociation barriers indicates that CO dissociation is favored over desorption on Pt _4_ Mo_3_ and Mo_7_.

The initial states of CO dissociation pathways on Pt_4_Mo_3_ and Mo_7_ are multifold tilted CO adsorption configurations where both C and O atoms are simultaneously bonded to Mo atoms. The energetical tendency of Mo atoms to form clusters instead of being distributed among Pt atoms leads to the formation of multifold Mo sites in the nanoparticle which seems to be a prerequisite for CO dissociation. However, these Mo clusters also prefer the higher coordinated inner sites in the nanoparticle. Steric effects make it difficult for CO to bind these Mo clusters in the center of nanoparticle if the Mo concentration in the nanoparticle is low. Structurally, Mo sites suitable for multifold CO adsorption, and, consequently energetically favorable CO dissociation reaction, are possible when Mo concentration reaches a certain limit in the mixed nanoparticles, as in the case of Pt_4_Mo_3_ at size 7.

CO adsorption energy is overall reduced by mixing of Pt and Mo. On Pt_5_Mo_2_ and Pt_4_Mo_3_, CO adsorption energies on the most stable adsorption sites are significantly lower than that of both Pt_7_ and Mo_7_. However, on Pt_4_Mo_3_ CO dissociation can produce C and O atoms that occupy the reactive sites on the nanoparticles and prevent other reactants to get adsorbed. Thus, dissociation does not offer a solution to the poisoning problem. In this respect, Pt_5_Mo_2_ is a promising composition with the highest resistance to the problem of CO poisoning. The findings can guide experiments on the synthesis of nanoparticles, including nanocatalysts suitable for applications containing CO. Although these findings are obtained with smaller sizes, they give hints for the design of larger Pt-Mo nanoparticles with enhanced catalytic properties.

Supplementary Materials
