# Supplementary material for: Effects of composition on catalytic activities of molybdenum doped platinum nanoparticles
Source: Turk J Chem. 2020 Aug 18;44(4):1016–30. doi: 10.3906/kim-2001-63 (PMC7751935; doi:10.3906/kim-2001-63)
Supplement: Supplementary file 1 — Supplementary Materials [file turkjchem-44-1016-sup001.pdf]

Supplemental material

Methodology employed to generate initial geometric coordinates of metal clusters

The initial geometric coordinates used to sample the potential energy surface of Pt_7 and Mo_7 configurations were mainly obtained from literature. Initial mixed structures were generated from these pure structures by replacing the symmetry-unique Pt (Mo) atoms in the Pt_7 (Mo_7) with Mo (Pt) atoms in a systematic fashion.

To further increase the chance of reaching the global minima, additional initial geometric coordinates were generated using simulated annealing simulations. Some of low energy structures obtained as above are used as initial structures of the pseudopotential plane-wave (PSPW) density functional theory Car-Parinello molecular dynamics simulated annealing method as implemented in NWChem. During the simulated annealing simulations, Nose-Hoover constant temperature is used. The clusters were first thermally equilibrated at 2000 K. They are then annealed for 12.09 ps with a time step of 5 a.u. Temperatures are scaled according to an exponential cooling schedule with a scaling time constant of 1ps. The metastable structures were then optimized with the PSPW level of theory. In the PSPW calculations, LDA was used as the exchange correlation functional. The boundary condition is free-space, and the simulation cell is aperiodic and cubic with a side length of 20 Å.

After the final optimizations of the geometries generated through the methodologies described above (using atomic basis sets similar to the rest of the work), close to 100 unique stable metal clusters at different compositions are obtained in total.

Optimized CO-$Pt_{7-n}Mo_n$ (n = 0–3,7) adsorption complexes

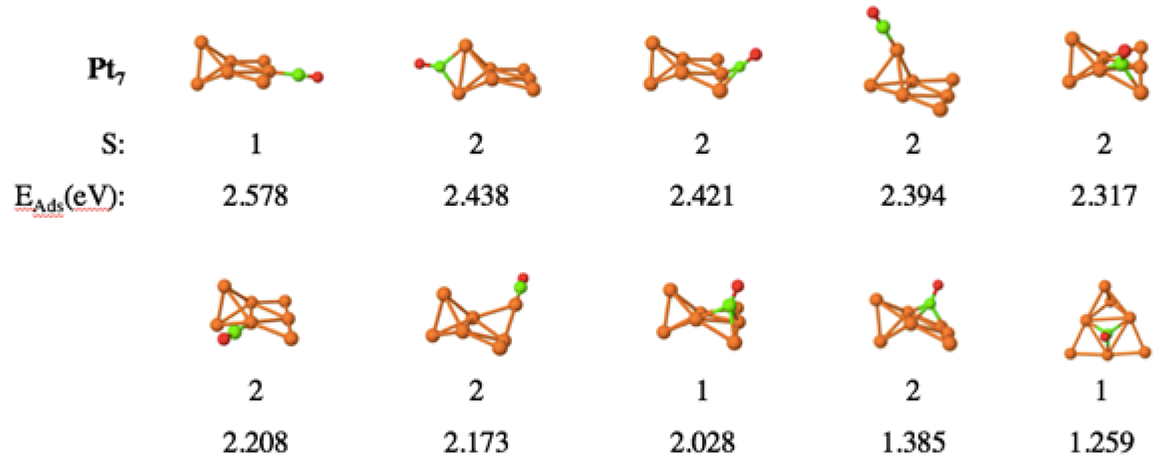

Figure S1. Stable CO-Pt_7 with spin states (S) and CO adsorption energies (E_{Ads}).

CO dissociation pathways of CO-$Pt_{7-n}Mo_n$ (n =0–3,7)

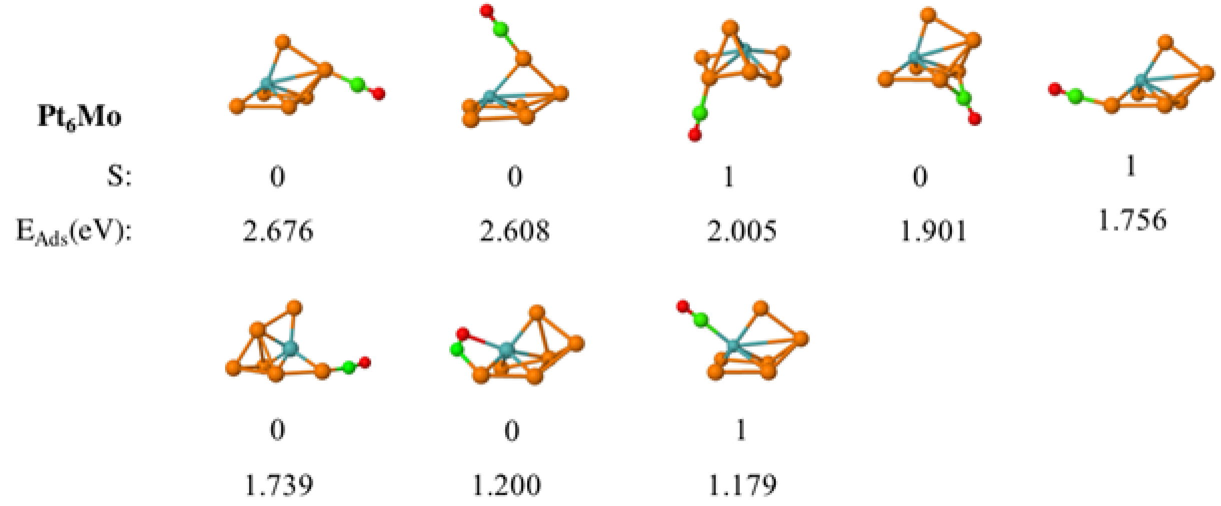

Figure S2. Stable CO-Pt_6Mo with spin states (S) and CO adsorption energies (E_{Ads}).

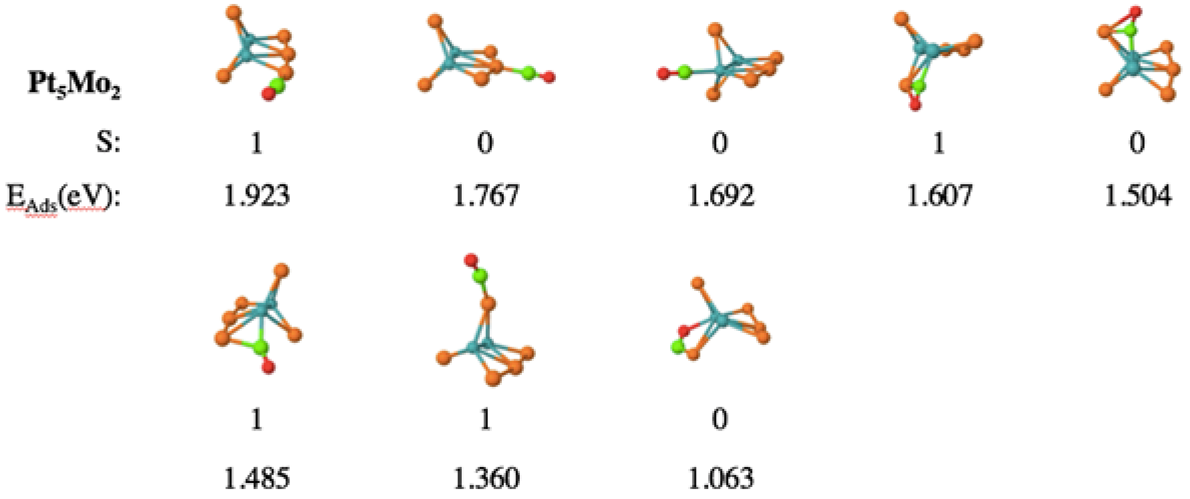

Figure S3. Stable CO-Pt_5Mo_2 with spin states (S) and CO adsorption energies (E_{Ads}).

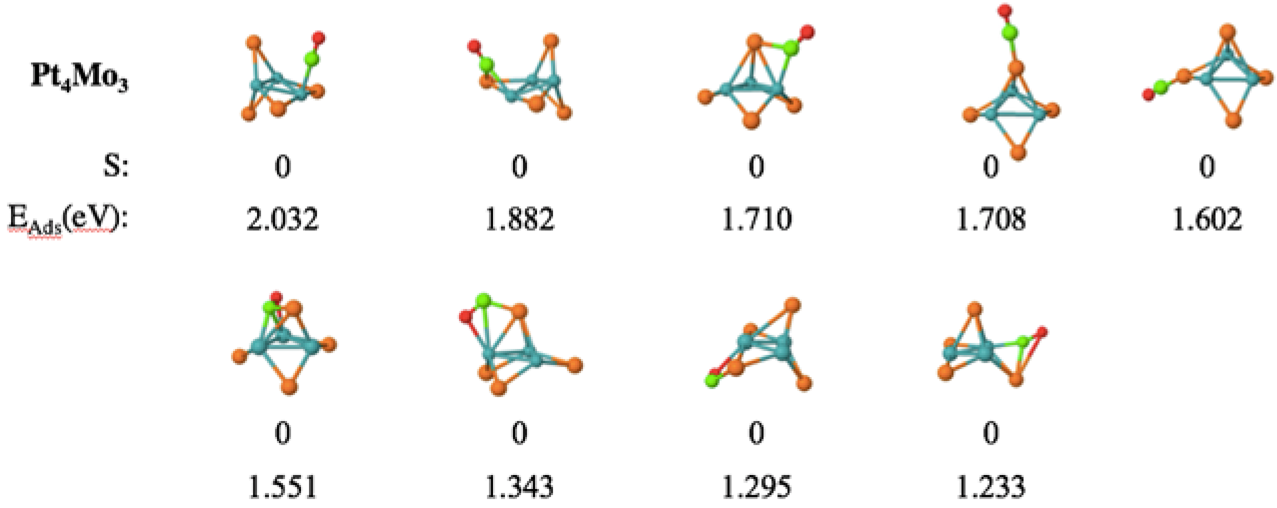

Figure S4. Stable CO-Pt_4Mo_3 with spin states (S) and CO adsorption energies (E_{Ads}).

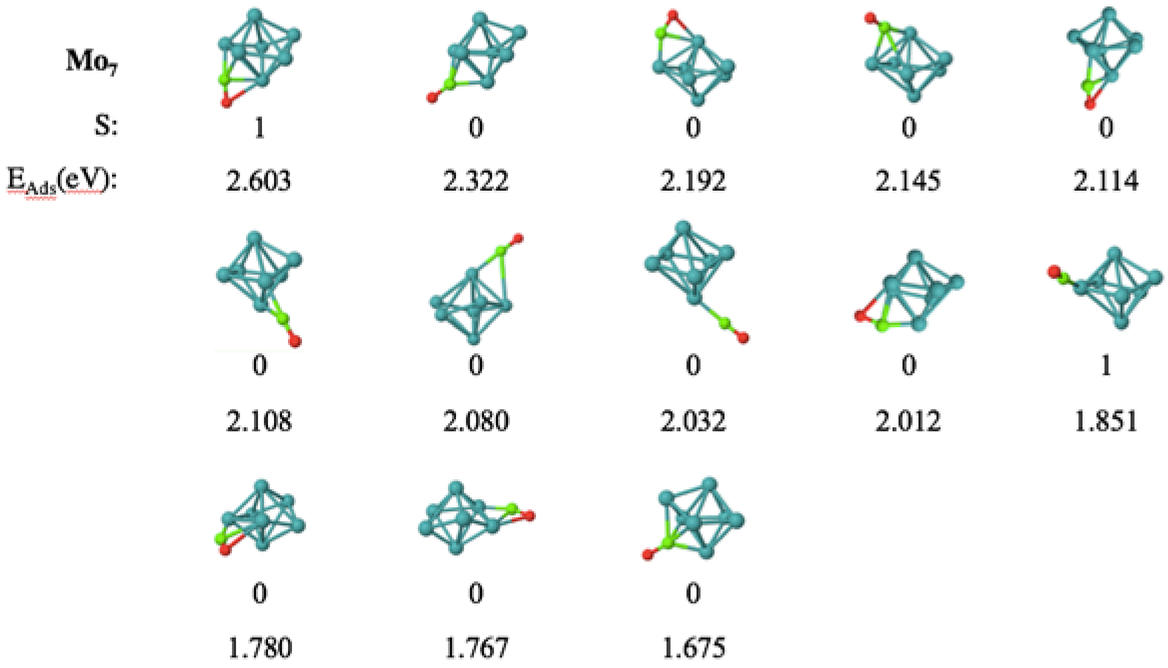

Figure S5. Stable CO-Mo_7 with spin states (S) and CO adsorption energies (E_{Ads}).

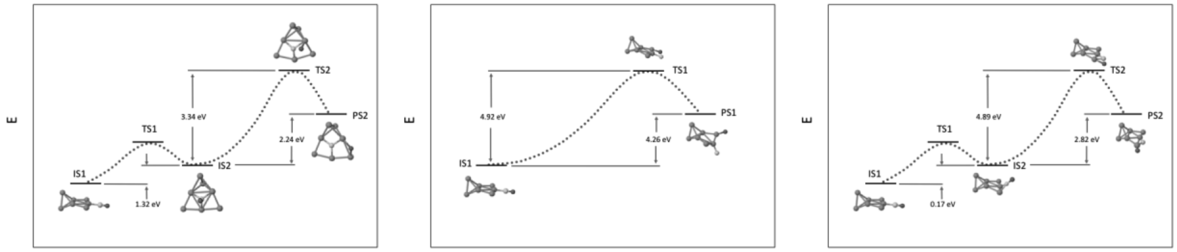

Figure S6. CO dissociation paths for CO-Pt_7. Energies of initial (IS), transition (TS) and product states (PS) are given. The dotted lines that connect IS, TS and PS are for visual purposes and do not represent real points in the potential energy surface.

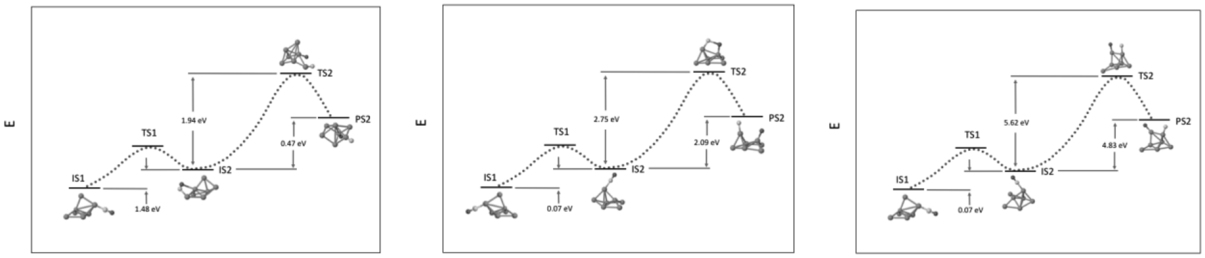

Figure S7. CO dissociation paths for CO-Pt_6Mo. Energies of initial (IS), transition (TS) and product states (PS) are given. The dotted lines that connect IS, TS and PS are for visual purposes and do not represent real points in the potential energy surface.

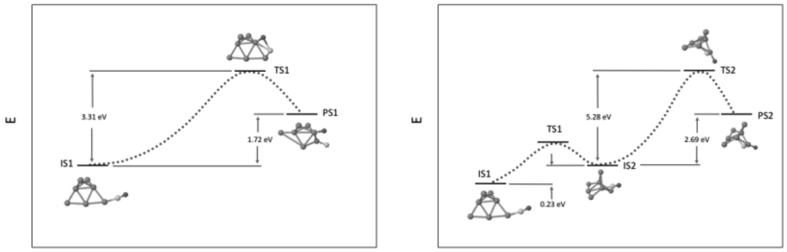

Figure S8. CO dissociation paths for CO-Pt_5Mo_2. Energies of initial (IS), transition (TS) and product states (PS) are given. The dotted lines that connect IS, TS and PS are for visual purposes and do not represent real points in the potential energy surface.

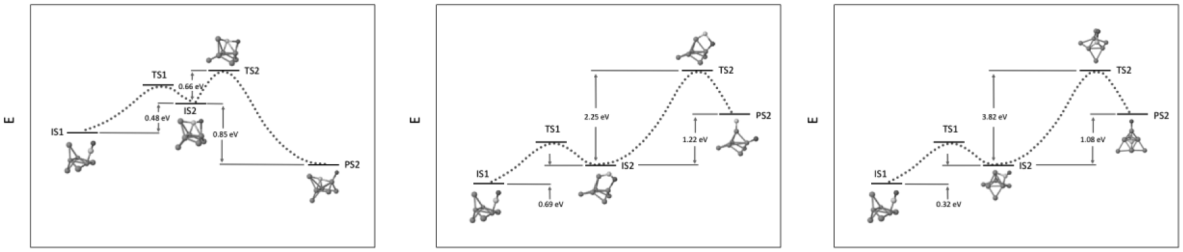

Figure S9. CO dissociation paths for CO-Pt_4Mo_3. Energies of initial (IS), transition (TS) and product states (PS) are given. The dotted lines that connect IS, TS and PS are for visual purposes and do not represent real points in the potential energy surface.

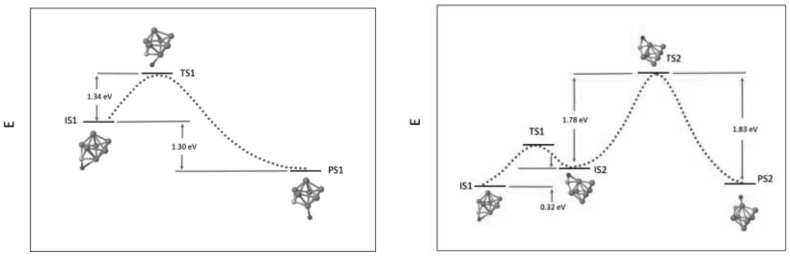

Figure S10. CO dissociation paths for CO-Mo_7. Energies of initial (IS), transition (TS) and product states (PS) are given. The dotted lines that connect IS, TS and PS are for visual purposes and do not represent real points in the potential energy surface.
